# Elevated CO_2_ Decouples the Short-Term Thermal Responses of Mesophyll and Stomatal Conductance, Enhancing Carbon Gain and Water Use at Moderate Heat but Failing at Extreme Temperatures

**DOI:** 10.3390/plants15182889

**Published:** 2026-09-21

**Authors:** Ruining Liu, Kai Jiang, Lehao Li, Jimei Han

**Affiliations:** National Key Laboratory for Development and Utilization of Forest Food Resources, Co-Innovation Center for Sustainable Forestry in Southern China, Nanjing Forestry University, Nanjing 210037, China; ningxiaorui@njfu.edu.cn (R.L.); kaijiang@njfu.edu.cn (K.J.); lehaoli@njfu.edu.cn (L.L.)

**Keywords:** *Liriodendron tulipifera* L., elevated CO_2_, short-term thermal response for photosynthesis, optimal temperature, mesophyll conductance, stomatal conductance, relative limitation analysis

## Abstract

Rising atmospheric CO_2_ concentration and global warming jointly affect plant photosynthesis and water use, but how elevated CO_2_ regulates photosynthetic short-term thermal response and its implications for leaf carbon-water balance remain poorly understood. We investigated the temperature responses (25–45 °C) of net photosynthetic rate (*A*_n_), intrinsic water use efficiency (iWUE), stomatal conductance (*g*_s_), and mesophyll conductance (*g*_m_) in *Liriodendron tulipifera* L., a fast-growing and economically important hardwood species sensitive to heat stress, under five CO_2_ concentrations (150–800 μmol mol^−1^), and quantified the partitioning of stomatal, mesophyll, and biochemical limitations. Elevated CO_2_ increased the optimal temperature (*T*_opt_) of *A*_n_, iWUE, and *g*_m_, but decreased that of *g*_s_, creating a divergent thermal response pattern that decoupled carbon acquisition from water loss. The upward shift in the *T*_opt_ of *g*_m_ enhanced CO_2_ supply to chloroplast carboxylation sites at high temperatures, sustaining higher carboxylation rates, indicating that improved mesophyll diffusion, rather than biochemical capacity, drove photosynthetic short-term thermal response. The downward shift in *T*_opt_ of *g*_s_ aligned with optimal stomatal behavior theory, promoting earlier stomatal closure to conserve water, which together with enhanced mesophyll diffusion resulted in a net improvement in water use efficiency below 40 °C. At 45 °C, however, mesophyll limitation (*l*_m_) became dominant (~70% of total limitation), indicating that elevated CO_2_ cannot prevent photosynthetic breakdown under extreme heat, and the water-saving benefit was lost. We suggest that terrestrial biosphere models incorporating separate temperature dependencies of *g*_m_ and *g*_s_ would improve predictions of tree carbon-water balance under future climates.

## 1. Introduction

Global warming and rising atmospheric CO_2_ concentrations represent two concurrent facets of climate change that impose profound pressures on plant carbon gain and water relations [1,2]. By the end of this century, under the very high-emission scenario (SSP5-8.5), global mean temperature is projected to rise by 3.3–5.7 °C, with CO_2_ levels reaching approximately 1135 ppm [3]. For plants, elevated CO_2_ offers a potential “fertilization effect” that enhances photosynthesis, yet this benefit is often counteracted by the negative impacts of heat stress on photosynthetic biochemistry and stomatal regulation [4,5]. Understanding how rising temperatures interact with CO_2_ concentration to shape the coordinated responses of carbon assimilation and transpiration is therefore essential for predicting plant performance, forest productivity, and ecosystem resilience in future climates.

The diffusion of CO_2_ from the atmosphere to the chloroplast carboxylation sites involves two major conductance components in series: stomatal conductance (*g*_s_), which regulates CO_2_ entry through the stomatal pore, and mesophyll conductance (*g*_m_), which governs CO_2_ transport from the substomatal cavity to the site of Rubisco carboxylation within the mesophyll [6,7,8,9,10]. While *g*_s_ has long been recognized as a primary limiter of photosynthesis, growing evidence indicates that *g*_m_ imposes a comparable and sometimes larger limitation, reducing the CO_2_ concentration at Rubisco by 20–40% relative to the substomatal value [11,12]. Moreover, *g*_m_ is not a fixed physical property but a dynamic physiological trait, influenced by carbonic anhydrase (CA) activity, aquaporin permeability, and membrane fluidity [11,13,14,15]. Consequently, both *g*_s_ and *g*_m_ contribute to the overall diffusional limitation of photosynthesis, and their coordinated regulation is critical for optimizing the trade-off between CO_2_ supply and water loss under fluctuating environments.

The response of photosynthesis to temperature is characteristically nonlinear, with net photosynthetic rate (*A*_n_) rising as temperature increases from cool to moderate levels due to faster enzymatic kinetics, but declining sharply when temperatures exceed the thermal optimum (*T*_opt_), largely because of Rubisco deactivation and reduced electron transport capacity [5,16,17]. This temperature dependence extends beyond biochemistry to the two major diffusional components, *g*_s_ and *g*_m_. Both *g*_s_ and *g*_m_ generally increase with rising temperature within the moderate range, facilitating greater CO_2_ supply to match the increased carboxylation demand [18,19]. However, as temperature continues to rise beyond the optimum, both conductances decline, *g*_s_ primarily through stomatal closure driven by increased leaf-to-air vapor pressure deficit (VPD) [20,21], and *g*_m_ through physical and biochemical changes in the mesophyll such as altered membrane permeability, CA activity, and cell wall properties [22]. Because *A*_n_, *g*_s_, and *g*_m_ all peak within a similar temperature range, their temperature responses are often treated as broadly covarying, with maximum photosynthetic rates achieved only when both stomatal and mesophyll CO_2_ supply remain sufficient to sustain carboxylation [23,24]. This coordination is intuitively appealing, as carbon assimilation requires an uninterrupted CO_2_ supply chain from the atmosphere to Rubisco; if any link, whether *g*_s_ or *g*_m_, fails prematurely at high temperature, photosynthesis will become CO_2_-limited.

Elevated CO_2_, however, may fundamentally alter this coordinated thermal landscape. At the biochemical level, increased CO_2_ availability as a substrate for Rubisco could enhance carboxylation rates at high temperatures, potentially raising the thermal optimum of photosynthesis [25,26]. Nevertheless, the temperature response of Rubisco carboxylation capacity (i.e., the maximum carboxylation rate-*V*_cmax_) is expected to be relatively conservative, as the catalytic properties of Rubisco are constrained by its evolutionary origin and structural stability [5,25]. At the diffusional level, because elevated CO_2_ steepens the CO_2_ gradient from the atmosphere to the leaf interior, optimal stomatal behavior theory predicts that plants should down-regulate *g*_s_ and close stomata earlier under high CO_2_ to minimize water loss [27,28,29,30,31]. At the mesophyll level, emerging evidence suggests that elevated CO_2_ may also enhance the thermal stability of *g*_m_, possibly through up-regulation of CA activity or adjustments in aquaporin expression that sustain CO_2_ transport across the mesophyll at higher temperatures [15,32]. Whether these opposing influences, enhanced carbon supply and mesophyll diffusion versus earlier stomatal closure, lead to a coordinated or divergent shift in the temperature optima of *g*_m_, *g*_s_ remains unknown. Furthermore, the potential divergence in *g*_m_ and *g*_s_ responses, if it occurs, would likely alter the partitioning among stomatal, mesophyll, and biochemical limitations across temperatures; yet how elevated CO_2_ reshapes this partitioning remains unexplored. In addition, heat stress can directly impair photosystem II photochemistry and electron transport [16,26], but whether elevated CO_2_ alleviates such photodamage is also uncertain.

However, to our knowledge, empirical evidence directly testing this divergent shift within a single experimental framework, using simultaneous measurements of *A*_n_, *g*_m_, and *g*_s_ across a wide temperature range under elevated CO_2_, remains remarkably scarce. This knowledge gap is especially critical for temperate forests, which experience broad seasonal and diurnal temperature fluctuations and are projected to face increasingly frequent heatwaves under future climate scenarios [33,34]. Most previous studies have focused separately on photosynthetic heat tolerance or stomatal responses, without quantifying how the temperature optima of *g*_m_ and *g*_s_ shift relative to each other under CO_2_ enrichment, nor how the partitioning of stomatal, mesophyll, and biochemical limitations shifts with temperature under elevated CO_2_. In contrast, the present study provides the first simultaneous measurements of *A*_n_, *g*_s_, and *g*_m_ across a wide temperature range under multiple CO_2_ concentrations, allowing us to directly test whether elevated CO_2_ induces a divergent shift in the *T*_opt_ of *g*_m_ versus *g*_s_ [6,11].

As one of the most important commercial hardwood species in eastern North America, *Liriodendron tulipifera* L. is highly valued for its rapid growth and high-quality timber, and it also exhibits exceptionally high carbon sequestration capacity [35,36,37]. Moreover, as a mesic species, it is particularly sensitive to heat stress, making it a suitable indicator for assessing the impacts of climate change on forest ecosystems [36,38]. Understanding how elevated CO_2_ and temperature affect its photosynthetic physiology therefore has both ecological and practical significance. In this study, we used *Liriodendron tulipifera* L. to systematically investigate the effects of elevated CO_2_ on photosynthetic heat acclimation and CO_2_ diffusion processes under controlled CO_2_ concentration and temperature conditions. The specific objectives of this study were: (1) to quantify the individual temperature responses of *A*_n_, *g*_s_, and *g*_m_ under both ambient and elevated CO_2_ concentrations; (2) to determine whether elevated CO_2_ induces a divergent shift in the *T*_opt_ of *g*_m_ versus *g*_s_; and (3) to evaluate the consequences of such divergent responses for leaf-level carbon-water balance across a thermally relevant range. By integrating simultaneous measurements of gas exchange and photosynthetic CO_2_ diffusion, we aim to provide a mechanistic understanding of how rising CO_2_ may reconfigure the thermal coordination between CO_2_ supply and demand, and to improve current predictions of tree physiological performance under future climate scenarios.

## 2. Results

### 2.1. The Temperature Response of Photosynthetic Parameters Under Different CO_2_ Concentrations

**Figure 1:**
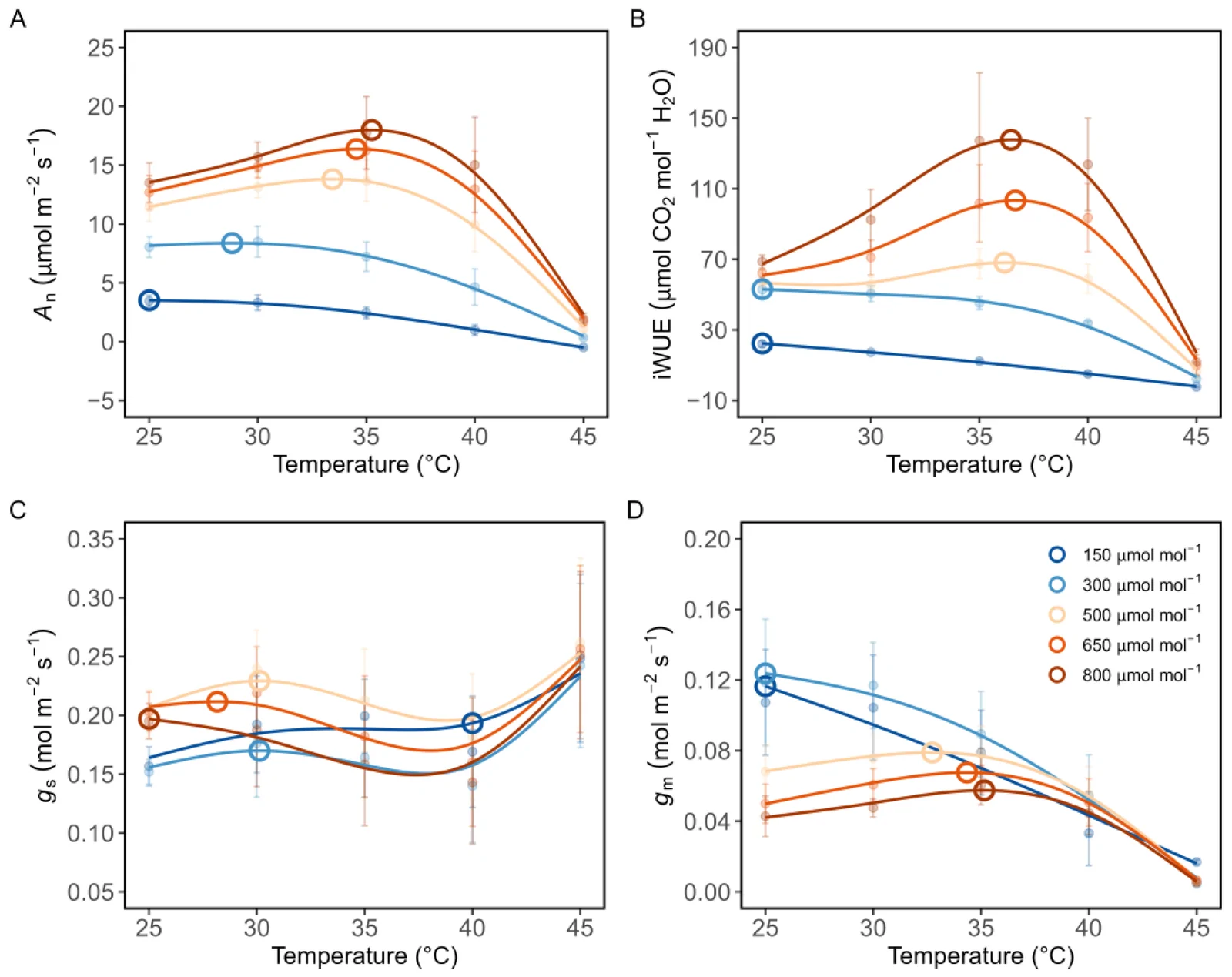
Responses of photosynthetic parameters to temperature across a range of CO_2_ concentrations (150, 300, 500, 650, and 800 μmol mol^−1^), measured at photosynthetically active radiation (PAR) of 1200 μmol m^−2^ s^−1^. (**A**) Net photosynthetic rate (*A*_n_); (**B**) intrinsic water use efficiency (iWUE, *A*_n_/*g*_s_); (**C**) stomatal conductance (*g*_s_); (**D**) mesophyll conductance (*g*_m_). Solid lines are GAMM-fitted curves for different parameters; solid dots are observed means for each temperature-C_a_ combination; vertical bars are standard errors (SE, n = 3). Hollow circles in *A*_n_, iWUE, *g*_s_, and *g*_m_ mark GAMM-estimated optimal temperatures (*T_opt_*), with 95% confidence intervals estimated by parametric bootstrap (Table 1); colors denote *C*_a_ concentrations.

**Figure 2:**
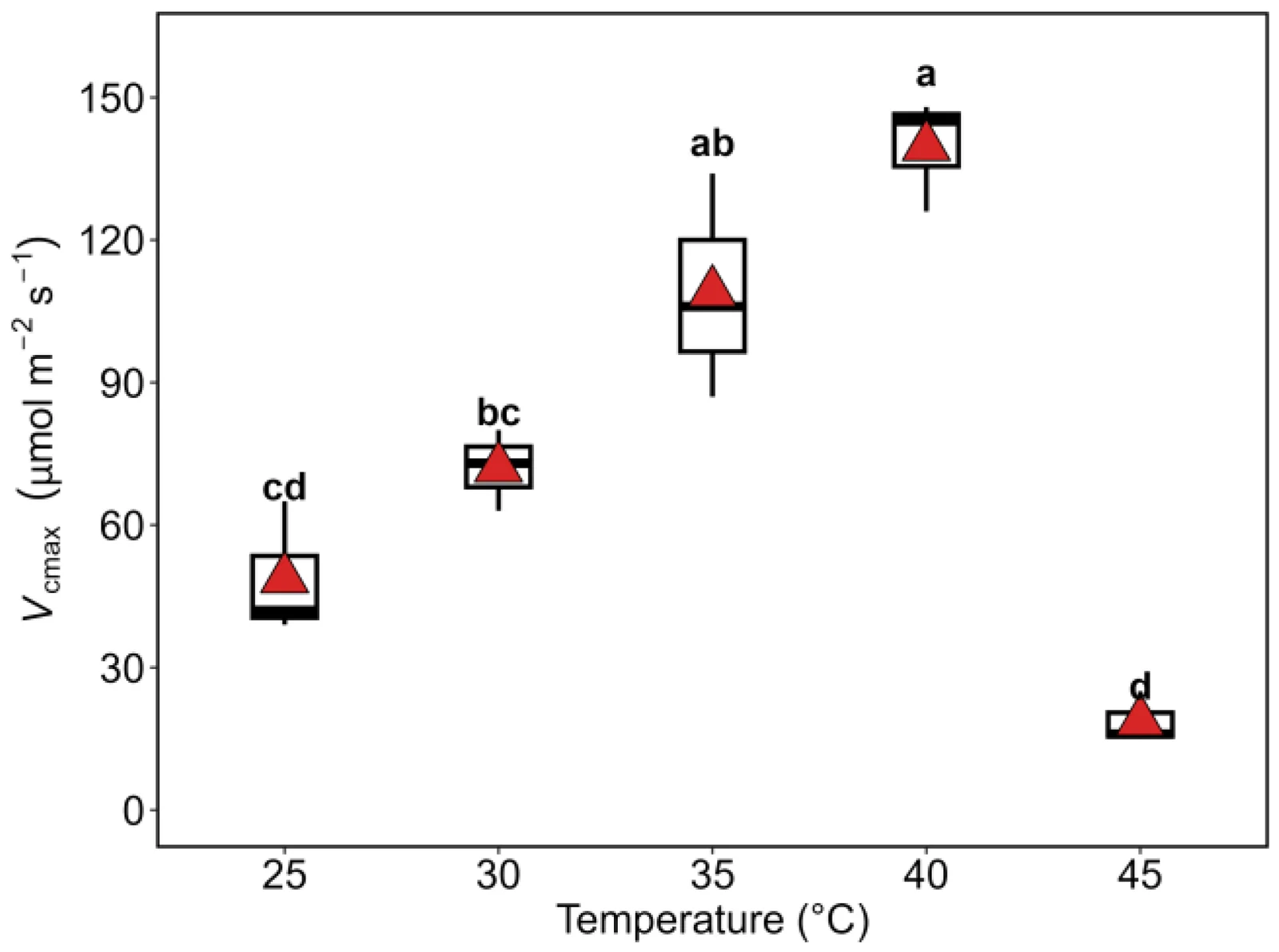
Temperature response of maximum carboxylation rate (*V*_cmax_). Boxplots display the interquartile range (IQR, 25th–75th percentile, white boxes) and median line. Red diamond markers represent mean values of three replicates. Statistical significance among temperature treatments was assessed using one-way analysis of variance (ANOVA), with the significance threshold set at *p* < 0.05. Sample size for each temperature: n = 3. Different lowercase letters (a–d) above the boxes indicate significant differences among temperature treatments based on post-hoc multiple comparison tests; treatments sharing the same letter are not significantly different.

To characterize the temperature responses of photosynthetic parameters under different CO_2_ concentrations, we first examined the patterns of *A*_n_, intrinsic water use efficiency (iWUE), *g*_s_, *g*_m_, and *V*_cmax_ across the temperature gradient. As shown in Figure 1A, *A*_n_ increased with rising CO_2_ concentration. Across all CO_2_ treatments, *A*_n_ first increased and then decreased with increasing temperature, and its optimal temperature shifted upward with elevated CO_2_ (see Table 1 for specific *T*_opt_ values). iWUE exhibited a unimodal response to temperature under elevated CO_2_ (>400 μmol mol^−1^), peaking at 35–40 °C and decreasing markedly thereafter. In contrast, under low CO_2_ (<400 μmol mol^−1^), iWUE declined monotonically with increasing temperature. Elevated CO_2_ partially mitigated the negative effects of heat stress on iWUE; however, this protective effect was lost at temperatures exceeding 40 °C (Figure 1B). The response of g_s_ to CO_2_ differed from that of *A*_n_ (Figure 1C). *g*_s_ first increased and then decreased with rising temperature; however, when temperature exceeded 40 °C, *g*_s_ increased again. Below 40 °C, the *T*_opt_ of *g*_s_ decreased with increasing CO_2_ concentration (Table 1). Moreover, the response of *g*_s_ to CO_2_ concentration varied with temperature. Below 35 °C, *g*_m_ decreased with increasing CO_2_ concentration; above 35 °C, the effect of CO_2_ on *g*_m_ diminished. Under 150 and 300 μmol mol^−1^ CO_2_, *g*_m_ declined slowly and then rapidly with increasing temperature. In contrast, under 500, 650, and 800 μmol mol^−1^ CO_2_, *g*_m_ first increased and then decreased with temperature (Figure 1D). The optimal temperature of *g*_m_ increased with rising CO_2_ concentration. *V*_cmax_ showed a clear temperature-dependent pattern, with values rising from 50 μmol m^−2^ s^−1^ at 25 °C to a maximum of 140 μmol m^−2^ s^−1^ at approximately 40 °C, followed by a sharp decline as temperature continued to increase (Figure 2).

**Figure 3:**
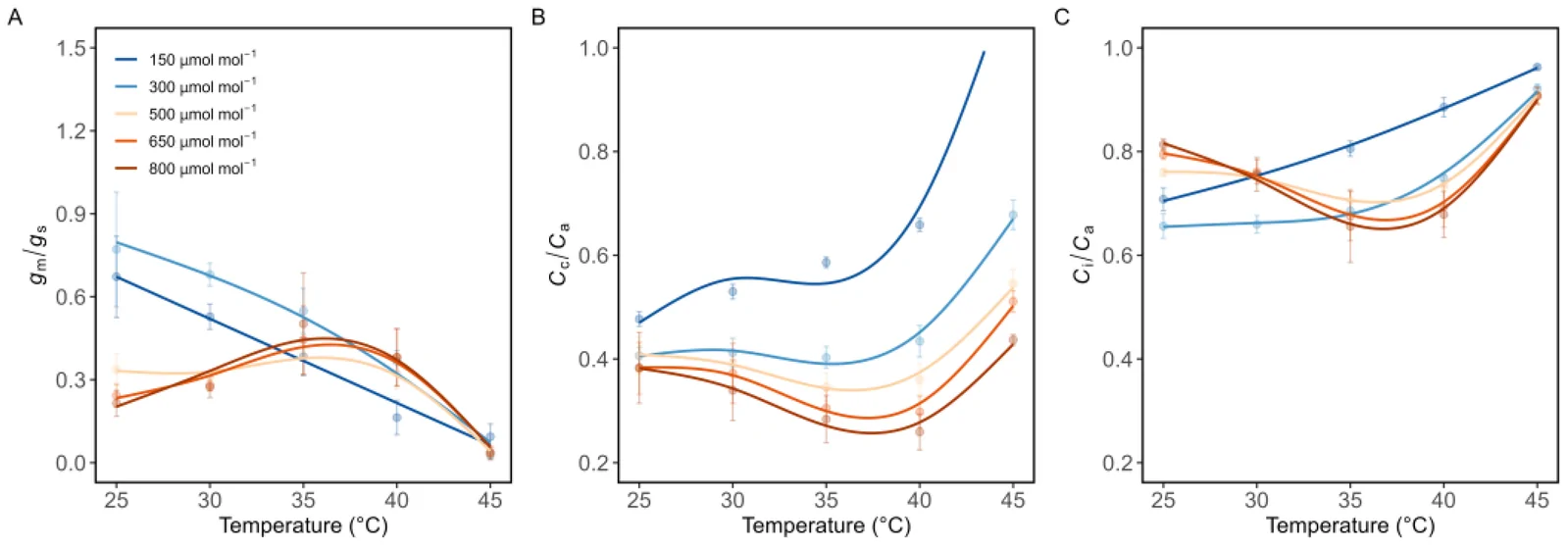
Temperature responses of CO_2_-diffusion-related ratios across a range of CO_2_ concentrations (150, 300, 500, 650, and 800 μmol mol^−1^), measured at a PAR of 1200 μmol m^−2^ s^−1^. (**A**) ratio of mesophyll to stomatal conductance (*g*_m_/*g*_s_); (**B**) ratio of chloroplastic to ambient CO_2_ concentration (*C*_c_/*C*_a_); (**C**) ratio of intercellular to ambient CO_2_ concentration (*C*_i_/*C*_a_). Solid lines are GAMM-fitted curves; solid dots are observed means for each temperature-*C*_a_ combination; vertical bars are SE (n = 3); different colors denote different CO_2_ concentrations as indicated in the legend. All GAMM smooth terms were tested for significance, with the significance threshold was *p* < 0.05.

The temperature response of ratio of mesophyll to stomatal conductance (*g*_m_/*g*_s_) followed a similar pattern to that of *g*_m_, except that the response curves under 500, 650, and 800 μmol mol^−1^ CO_2_ largely overlapped (Figure 3A). Ratio of chloroplastic to ambient CO_2_ concentration (*C*_c_/*C*_a_) decreased with increasing CO_2_ concentration. Below 30 °C, *C*_c_/*C*_a_ remained relatively stable with temperature; it declined between 30 and 40 °C and increased again above 40 °C (Figure 3B). Under high CO_2_ concentrations (500, 650, and 800 μmol mol^−1^), ratio of intercellular to ambient CO_2_ concentration (*C*_i_/*C*_a_) first decreased and then increased with temperature; under low CO_2_ concentrations (150 and 300 μmol mol^−1^), it showed a monotonic increasing trend with temperature (Figure 3C).

To further evaluate the effects of temperature, CO_2_ concentration, and their interaction on photosynthetic traits, we conducted two-way ANOVA for each parameter. Temperature had significant effects on most parameters (*p* < 0.05), while CO_2_ concentration significantly affected *A*_n_, *g*_m_, iWUE, *g*_m_/*g*_s_, *C*_c_/*C*_a_, and *C*_i_/*C*_a_ (*p* < 0.05). Significant temperature × CO_2_ interactions were observed for *A*_n_, *g*_m_, iWUE, *g*_m_/*g*_s_, *C*_c_/*C*_a_, and *C*_i_/*C*_a_ (*p* < 0.05), indicating that the response of these traits to temperature was modulated by CO_2_ concentration (Table S2).

Model diagnostics confirmed that the GAMM adequately described the temperature responses. Because a separate GAMM was fitted for each combination of photosynthetic parameter and CO_2_ treatment, we present a representative example. The residual diagnostics for the GAMM fitted to *A*_n_ under Ca = 150 μmol mol^−1^ are shown in Figure S2. No obvious systematic pattern was observed between residuals and fitted values, and the points in the Q-Q plot generally followed the diagonal line, indicating that the model residuals approximately satisfied the assumptions of normality and homoscedasticity.

### 2.2. Photosynthetic Limitation Analyses

**Figure 4:**
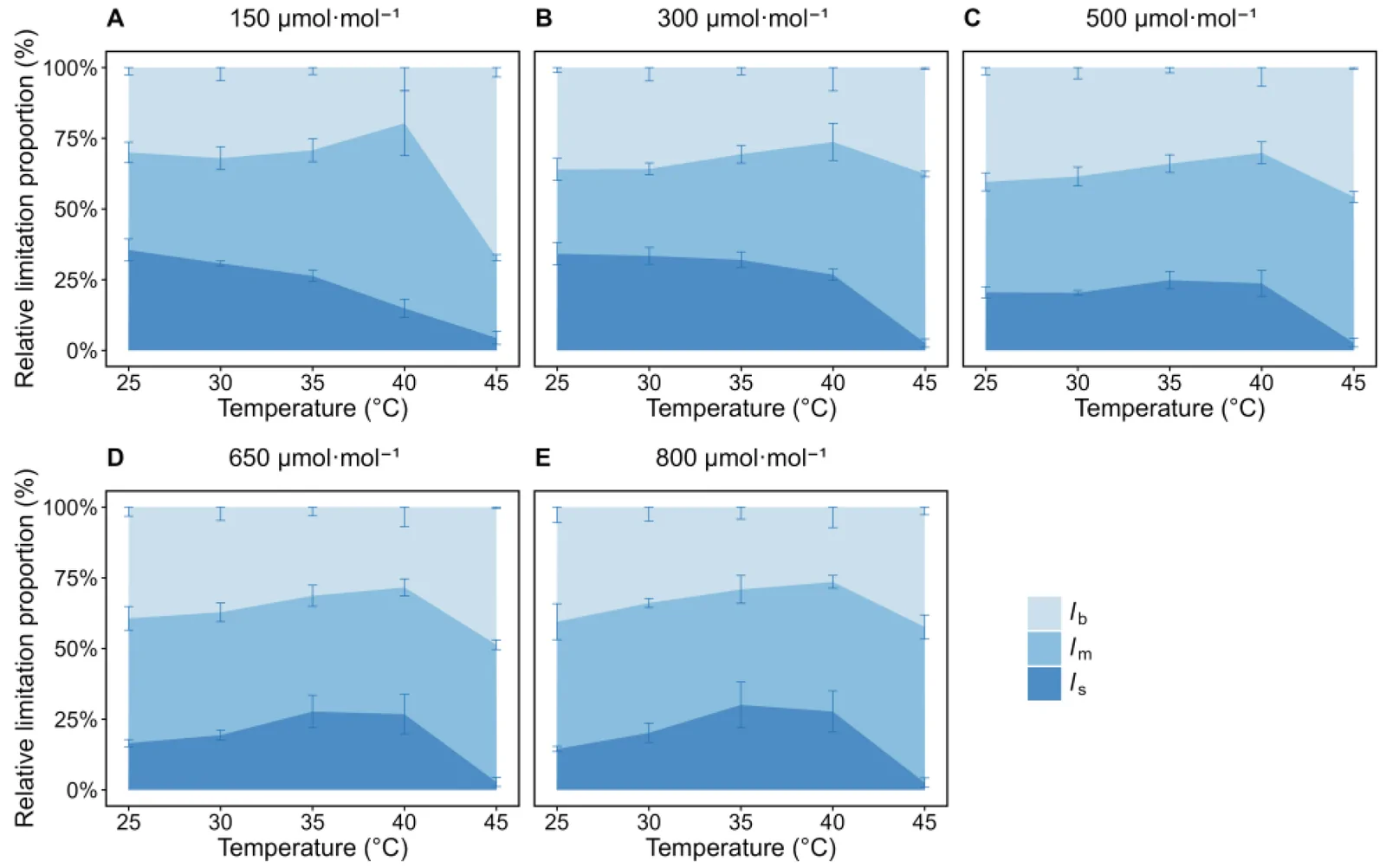
Temperature-driven shifts in the relative contributions of photosynthetic limitation components (*l*_b_: biochemical limitation; *l*_m_: mesophyll limitation; *l*_s_: stomatal limitation) under different CO_2_ concentrations at a PAR of 1200 μmol m^−2^ s^−1^. (**A**) Relative limitation proportions at 150 μmol mol^−1^ CO_2_; (**B**) at 300 μmol mol^−1^ CO_2_; (**C**) at 500 μmol mol^−1^ CO_2_; (**D**) at 650 μmol mol^−1^ CO_2_; (**E**) at 800 μmol mol^−1^ CO_2_. The y-axis represents the relative contribution (%) of each limitation component, with the sum of *l*_b_ + *l*_m_ + *l*_s_ = 100%. Vertical error bars represent standard errors (SE, n = 3).

Under low CO_2_ concentrations (150 and 300 μmol mol^−1^), stomatal limitation (*l*_s_) remained relatively stable or declined slightly with increasing temperature, but decreased sharply when temperature exceeded 40 °C. Under high CO_2_ concentrations (500, 650, and 800 μmol mol^−1^), *l*_s_ first increased with temperature, leveled off around 35 °C, and then declined rapidly above 40 °C. Mesophyll limitation (*l*_m_) increased consistently with rising temperature across all CO_2_ treatments, reaching its maximum at 45 °C. Biochemical limitation (*l*_b_) decreased gradually with increasing temperature. However, above 40 °C, *l*_b_ either leveled off or increased to varying degrees, with the most pronounced increase observed under low CO_2_ concentrations (Figure 4).

### 2.3. Temperature Responses of Chlorophyll Fluorescence Parameters Under Different CO_2_ Concentrations

**Figure 5:**
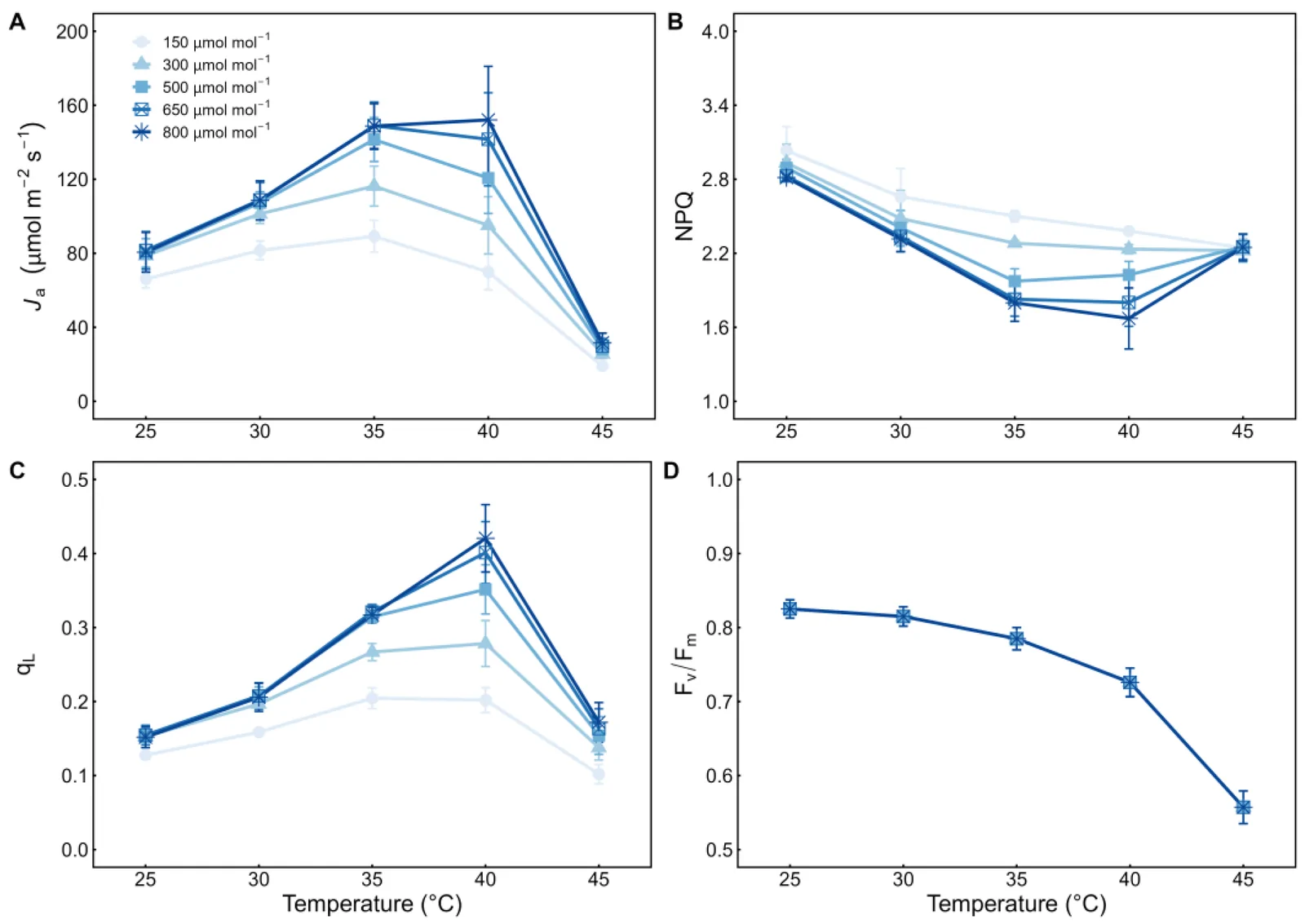
Temperature responses of chlorophyll fluorescence parameters under different CO_2_ concentrations (150, 300, 500, 650, and 800 μmol mol^−1^), measured at a PAR of 1200 μmol m^−2^ s^−1^. (**A**) Electron transport rate (*Jₐ*); (**B**) non-photochemical quenching (NPQ); (**C**) fraction of open PSII reaction center (*q*_L_); (**D**) maximum photochemical quantum efficiency of PSII (*F*_v_/*F*_m_). Vertical error bars represent standard errors (SE, n = 3). Different colors denote different CO_2_ concentrations as indicated in the legend. Statistical significance among temperature treatments was assessed using two-way analysis of variance (ANOVA), with the significance threshold was *p* < 0.05.

Under different CO_2_ concentrations, electron transport rate (*J*_a_) initially increased and then decreased with rising temperature, peaking at 35–40 °C, with overall enhancement under elevated CO_2_ (Figure 5A). Non-photochemical quenching (NPQ) generally declined with increasing temperature, reaching its minimum at 35–40 °C before rising again across all CO_2_ concentrations (Figure 5B). The low-CO_2_ treatment exhibited consistently higher NPQ levels, whereas the high-CO_2_ treatment showed a relatively greater decline. Fraction of open PSII reaction center (*q*_L_) continuously increased with rising temperature and reached its maximum at 40 °C (Figure 5C); at 45 °C, *q*_L_ decreased markedly across all CO_2_ concentrations, while elevated CO_2_ generally increased q_L_. With rising temperature, maximum photochemical quantum efficiency of PSII (*F*_v_/*F*_m_) exhibited a continuous declining trend (Figure 5D): the decrease was relatively gradual from 25 to 35 °C, but accelerated notably after 35 °C, reaching its lowest value at 45 °C. It should be noted that *F*_v_/*F*_m_ values at non−25 °C temperatures were estimated using published correction equations, as Minimum (*F*_o_) and maximum (*F*_m_) chlorophyll fluorescence were directly measured only at 25 °C. Furthermore, the 45 °C values were obtained after sequential exposure to lower temperatures; thus, the observed responses at this extreme temperature likely reflect cumulative heat stress rather than the direct effect of 45 °C per se.

### 2.4. Multivariate Relationships Among Photosynthetic Physiological Traits

**Figure 6:**
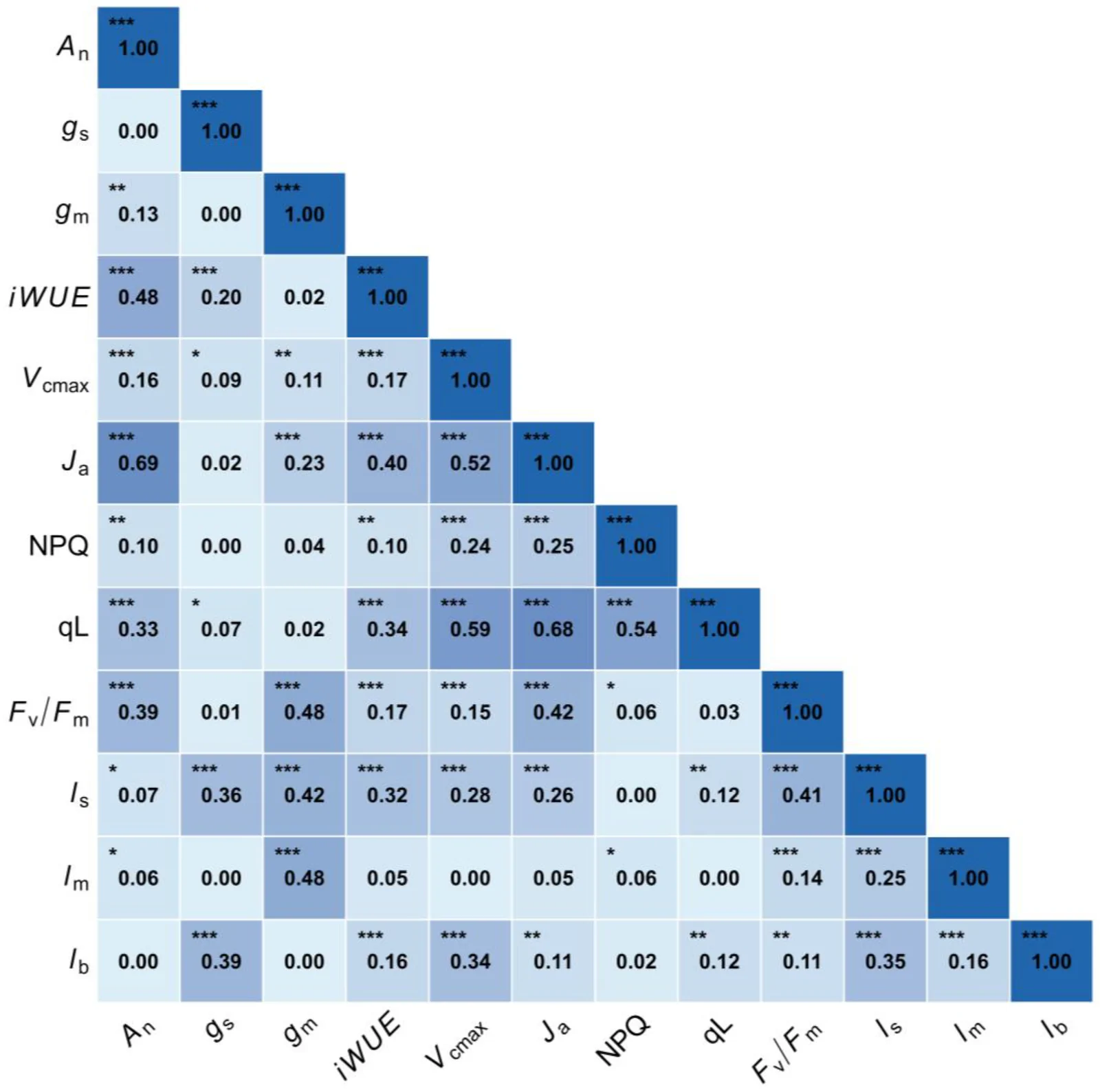
Correlation heatmap of photosynthetic parameters. Values within the boxes represent coefficients of determination (R^2^), and the color gradient indicates the strength of R^2^, with higher R^2^ values shown in darker colors, indicating stronger correlations. ***, **, and * denote significance at *p* < 0.001, *p* < 0.01, and *p* < 0.05, respectively. *A*_n_: net photosynthetic rate (μmol m^−2^ s^−1^); *g*_s_: stomatal conductance (mol m^−2^ s^−1^); *g*_m_: mesophyll conductance (mol m^−2^ s^−1^); iWUE: intrinsic water use efficiency (μmol CO_2_·mol^−1^ H_2_O); *V*_cmax_: Maximum carboxylation rate of Rubisco (μmol m^−2^ s^−1^); *J*_a_: Apparent electron transport rate (μmol m^−2^ s^−1^); NPQ: Non-photochemical quenching; *q*_L_: Fraction of open PSII reaction centers; *F*_v_/*F*_m_: Maximum quantum efficiency of PSII; *l*_s_: Stomatal limitation; *l*_m_: Mesophyll limitation; *l*_b_: Biochemical limitation.

**Figure 7:**
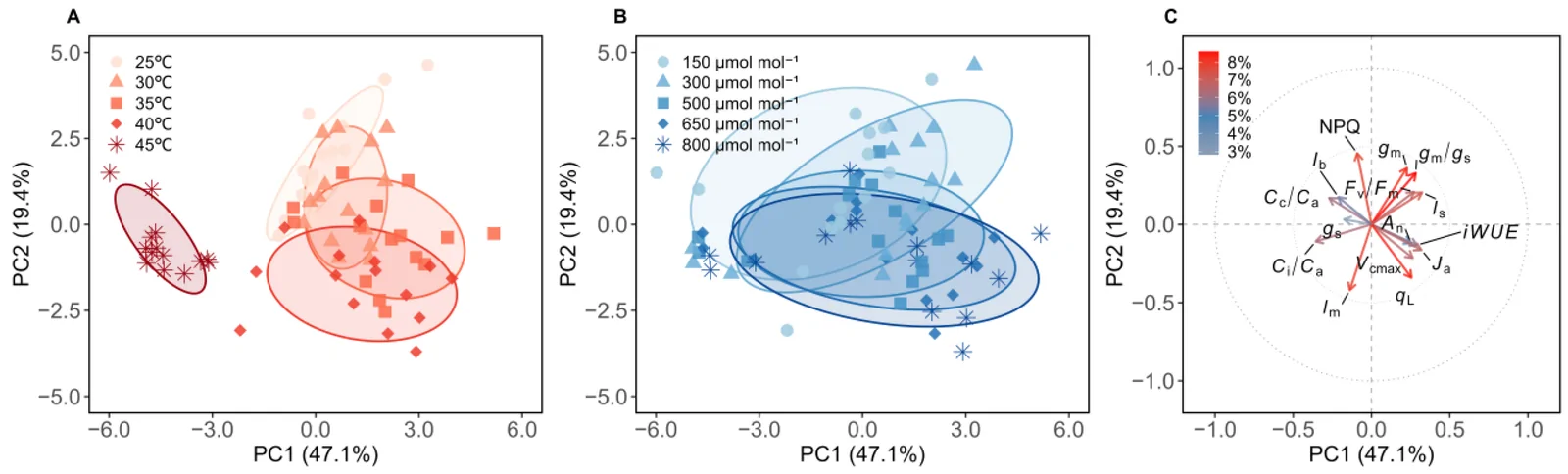
Principal component analysis (PCA) of photosynthetic and photochemical parameters across temperature and CO_2_ treatments. (**A**) Score plot colored by temperature (25, 30, 35, 40, and 45 °C). (**B**) Score plot colored by CO_2_ concentration (150, 300, 500, 650, and 800 μmol mol^−1^). (**C**) Loading plot showing the contribution of each parameter to PC1 and PC2; the color gradient indicates each variable’s contribution (%) to the principal components, and arrows denote the direction and magnitude of the loadings. Ellipses in (**A**,**B**) represent 95% confidence regions for each group. PC1 and PC2 explained 47.1% and 19.4% of the total variance, respectively. *A*_n_: net photosynthetic rate (μmol m^−2^ s^−1^); *g*_s_: stomatal conductance (mol m^−2^ s^−1^); *g*_m_: mesophyll conductance (mol m^−2^ s^−1^); iWUE: intrinsic water use efficiency (μmol CO_2_·mol^−1^ H_2_O); *V*_cmax_: Maximum carboxylation rate of Rubisco (μmol m^−2^ s^−1^); *J*_a_: Apparent electron transport rate (μmol m^−2^ s^−1^); NPQ: Non-photochemical quenching; *q*_L_: Fraction of open PSII reaction centers; *F*_v_/*F*_m_: Maximum quantum efficiency of PSII; *l*_s_: Stomatal limitation; *l*_m_: Mesophyll limitation; *l*_b_: Biochemical limitation; *C*_c_/*C*_a_: ratio of chloroplastic to ambient CO_2_ concentration; *C*_i_/*C*_a_: ratio of intercellular to ambient CO_2_ concentration.

Correlation analysis revealed distinct associations among the measured photosynthetic traits (Figure 6). *A*_n_ was significantly correlated with *J*_a_ (R^2^ = 0.69), iWUE (R^2^ = 0.48), *F*_v_/*F*_m_ (R^2^ = 0.39), *q*_L_ (R^2^ = 0.33), *V*_cmax_ (R^2^ = 0.16), and *g*_m_ (R^2^ = 0.13), indicating that photosynthetic performance was associated with variation in electron transport, intrinsic water use efficiency, photochemical characteristics, biochemical capacity, and mesophyll conductance. In contrast, *A*_n_ was not significantly correlated with *g*_s_ (R^2^ = 0.00), *l*_b_ (R^2^ = 0.00). The lack of a significant correlation between *A*_n_ and *g*_s_ may partly reflect the integration of all temperature treatments, because the temperature responses of stomatal conductance and photosynthetic assimilation were not necessarily synchronized across the full temperature range. Among the remaining traits, significant correlations were observed between *q*_L_ and both *J*_a_ (R^2^ = 0.68) and *V*_cmax_ (R^2^ = 0.59), between *q*_L_ and NPQ (R^2^ = 0.54), between *g*_m_ and both *F*_v_/*F*_m_ and *l*_m_ (R^2^ = 0.48), and between *J*_a_ and *V*_cmax_ (R^2^ = 0.52). The PCA further summarized the multivariate response patterns across temperature and CO_2_ treatments (Figure 7). PC1 and PC2 explained 47.1% and 19.4% of the total variance, respectively, together accounting for 66.5% of the overall variation. The temperature treatments showed a clear gradient primarily along PC1, with the 45 °C treatment clearly separated from the 25–40 °C treatments, whereas the CO_2_ treatments exhibited largely overlapping distributions (Figure 7A,B). This indicates that temperature, rather than CO_2_ concentration, was the dominant axis of multivariate variation in photosynthetic traits. The loading plot showed that PC1 was mainly loaded by *J*_a_, *q*_L_, *F*_v_/*F*_m_, *A*_n_, and iWUE, whereas NPQ loaded in the opposite direction (Figure 7C), consistent with the correlation patterns described above. These loadings indicate coordinated contributions of carbon assimilation, CO_2_ diffusion, and photochemical parameters to the observed multivariate response.

## 3. Discussion

### 3.1. Elevated CO_2_ Divergently Shifts the Thermal Optima of g_m_ and g_s_, Enhancing Photosynthetic Heat Tolerance While Improving Intrinsic Water Use Efficiency

Our results showed that elevated CO_2_ significantly increased the *T*_opt_ of *A*_n_ and *g*_m_, but decreased that of *g*_s_, creating a divergent thermal response pattern in which carbon acquisition and water loss became thermally decoupled (Figure 1). The upward shift in the *T*_opt_ of *g*_m_ allowed higher CO_2_ diffusion capacity to be maintained at elevated temperatures, increasing the CO_2_ concentration at the chloroplast carboxylation sites. This increase enhanced substrate availability for Rubisco carboxylation while suppressing photorespiratory competition, thereby sustaining a higher carboxylation rate at elevated temperatures [4,5]. In contrast to the pronounced upward shift in the *T*_opt_ of *g*_m_, the *T*_opt_ of *V*_cmax_ is expected to remain largely invariant with short-term CO_2_ elevation. This contrasting pattern suggests that the enhanced thermal tolerance of photosynthesis under elevated CO_2_ was driven primarily by improved mesophyll CO_2_ diffusion capacity, rather than by changes in biochemical capacity per se. As the CO_2_ diffusion capacity within the mesophyll, *g*_m_ determines the efficiency of CO_2_ transport from intercellular airspaces to the chloroplast carboxylation sites, and its variation is governed by multiple factors including cell wall thickness, liquid-phase diffusion pathways, carbonic anhydrase activity, and aquaporin regulation [6,39,40]. Previous studies have shown that elevated CO_2_ can induce adjustments in mesophyll anatomy and aquaporin expression, thereby enhancing CO_2_ supply to chloroplasts [11,41]. Thus, the elevated *T*_opt_ of *g*_m_ observed in our study likely reflects an enhanced short-term thermal response of the mesophyll diffusion system under elevated CO_2_. The close correspondence between the *T*_opt_ shifts of *A*_n_ and *g*_m_ further supports the interpretation that improved CO_2_ supply to the chloroplasts, rather than changes in carboxylation capacity per se, drove the enhanced thermal tolerance of photosynthesis.

In stark contrast to the rightward shift of *g*_m_, the *T*_opt_ of *g*_s_ shifted leftward under elevated CO_2_ (Table 1). This finding is consistent with the classic optimal stomatal behavior theory [27], which posits that stomata are regulated to maximize the ratio of carbon assimilation to transpirational water loss. Under elevated CO_2_, the steeper CO_2_ gradient from the atmosphere to the leaf interior allows the same *A*_n_ to be achieved at a lower *g*_s_ [30,42]. When extended to temperature responses, this optimization framework predicts that the temperature at which *g*_s_ optimization occurs should also shift downward: under high CO_2_, plants can achieve their optimal iWUE at lower temperatures, allowing them to close stomata earlier and conserve water while relying on enhanced *g*_m_ to compensate for reduced stomatal CO_2_ supply [27,43,44,45]. Our results provide direct experimental support for this prediction, demonstrating that elevated CO_2_ not only reduces the absolute magnitude of *g*_s_ but also lowers the thermal threshold for stomatal closure.

The opposing temperature responses of *g*_m_ and *g*_s_ observed here have important implications for leaf-level carbon-water balance. By shifting *T*_opt_ of *g*_m_ upward (enhancing CO_2_ supply to chloroplasts at high temperatures) and *T*_opt_ of *g*_s_ downward (promoting earlier stomatal closure to conserve water), elevated CO_2_ effectively decouples the temperature dependencies of carbon acquisition and water loss. The correlation analysis provided additional support for the coordinated variation among photosynthetic carbon assimilation, electron transport, and related physiological processes. *A*_n_ was significantly correlated with *J*_a_, iWUE, *F*_v_/*F*_m_, *q*_L_, *V*_cmax_, and *g*_m_, indicating that photosynthetic carbon assimilation was associated with changes in electron transport, intrinsic water-use efficiency, photochemical characteristics, biochemical capacity, and mesophyll conductance (Figure 6). In contrast, *A*_n_ was not significantly correlated with *g*_s_ or *l*_b_. The absence of a significant correlation between *A*_n_ and *g*_s_ may partly reflect the integration of all temperature treatments, because the temperature responses of stomatal conductance and photosynthetic assimilation were not necessarily synchronized across the full temperature range. Other significant correlations were observed among *q*_L_, *J*_a_, *V*_cmax_, and NPQ, as well as between *g*_m_ and *F*_v_/*F*_m_, *l*_m_, and *J*_a_ and *V*_cmax_. These relationships suggest that photosynthetic performance was associated with coordinated changes in CO_2_ diffusion, biochemical capacity, electron transport, and photochemical regulation. These relationships are consistent with the temperature-response patterns observed in the GAMM analyses and limitation analysis, further supporting the interpretation that coordinated changes in CO_2_ diffusion and biochemical processes contribute to the temperature dependence of photosynthesis. This decoupling resulted in a net enhancement of iWUE under elevated CO_2_, consistent with previous studies that have reported improved iWUE under CO_2_ enrichment [46,47]. Notably, this benefit was most pronounced at moderately elevated temperatures (30–40 °C), precisely the range where plants are most likely to experience heat stress under future climate scenarios.

### 3.2. Elevated CO_2_ Fails to Alleviate Photosynthetic Breakdown at 45 °C Due to Mesophyll Diffusion and Photochemical Impairment

Although elevated CO_2_ raised the thermal optima of both *A*_n_ and *g*_m_, its mitigating effect on extreme heat stress was clearly limited. At 45 °C, we observed a rapid decline in *l*_s_, whereas *l*_m_ emerged as the dominant constraint, accounting for approximately 70% of the total limitation (Figure 4). This interpretation is further supported by the *C*_c_/*C*_a_ data (Figure 3B), which showed that the CO_2_ concentration at the chloroplastic carboxylation site declined sharply at 45 °C, even under elevated CO_2_, indicating that CO_2_ transport to Rubisco was severely impeded regardless of external CO_2_ supply. These findings are consistent with previous studies showing that under severe heat stress, the capacity for internal CO_2_ diffusion toward chloroplasts becomes the key constraining process for photosynthesis [18], and they extend these earlier observations by demonstrating that this shift from stomatal to mesophyll limitation occurs across all CO_2_ conditions. The underlying mechanisms are well documented: extreme high temperatures can reduce *g*_m_ by destabilizing mesophyll cell membranes, decreasing aquaporin activity, and compromising chloroplast structural integrity, all of which restrict CO_2_ access to carboxylation sites [6,18,48]; in addition, the activity of carbonic anhydrase, which facilitates CO_2_ transport across the mesophyll, is temperature-sensitive and may decline under extreme heat. These temperature-induced changes in mesophyll function were severe enough to override any potential benefit from elevated CO_2_, indicating that CO_2_ enrichment cannot prevent the fundamental breakdown of mesophyll diffusion at extreme temperatures.

The sharp decline in photosynthetic capacity at 45 °C was accompanied by a pronounced decrease in estimated *F*_v_/*F*_m_ (Figure 5D), suggesting possible PSII impairment. This apparent photochemical limitation, together with reduced *q*_L_ and elevated NPQ, implies that the photosynthetic apparatus was functionally constrained at extreme temperatures. The failure of CO_2_ enrichment to sustain photosynthesis at 45 °C thus likely reflects photochemical capacity limitations rather than CO_2_ supply alone. However, because the *F*_v_/*F*_m_ values at non-25 °C temperatures were derived from published equations rather than measured directly in this study, these interpretations remain indicative and await direct experimental confirmation under the actual temperature regimes.

One notable exception was the increase in *g*_s_ when temperature exceeded 40 °C (Figure 1C). This anomalous rise, observed across all CO_2_ treatments, likely reflects stomatal dysfunction under extreme heat rather than an adaptive response, as membrane destabilization and impaired guard cell metabolism may lead to passive stomatal opening [49]. Alternatively, the elevated VPD at 40–45 °C may have driven passive hydraulic opening, as stomata lose the ability to maintain turgor-driven closure under extreme evaporative demand [50]. Although relative humidity was held constant in our experimental design, the concomitant rise in VPD with increasing temperature (from 1.37 kPa at 25 °C to 3.32 kPa at 45 °C; Table S1) may have contributed to the observed decline in *g*_s_, particularly at temperatures above 35 °C. Thus, the temperature response of *g*_s_ reported here reflects the combined effects of temperature and VPD, rather than temperature alone. This interpretation is supported by the concurrent decline in *A*_n_ and *g*_m_ at 45 °C, indicating that the *g*_s_ increase did not translate into enhanced photosynthesis [51].

Nevertheless, it is important to acknowledge that the 45 °C responses presented here may have been influenced by the cumulative thermal history of the leaves. Because measurements were conducted following sequential exposure to 25, 30, 35, and 40 °C, the observed decline at 45 °C could partly reflect progressive heat damage that had already been initiated at lower temperatures, rather than the instantaneous effect of 45 °C alone. This stepwise warming regime, however, realistically mimics natural heatwave events, during which plants experience gradual temperature increases over consecutive days rather than abrupt thermal shocks.

In addition, we acknowledge that anatomical and morphological traits, such as stomatal density and aperture, leaf area, leaf thickness, and specific leaf area, would provide valuable complementary evidence for the mechanistic understanding of plant responses to elevated CO_2_ and temperature. However, given that the present experiment was short-term in nature, such structural traits were unlikely to change appreciably during the measurement period, and they were therefore not assessed in this study. Future long-term temperature response experiments incorporating these traits would further strengthen our understanding of the structural underpinnings of photosynthetic acclimation under elevated CO_2_ and temperature.

## 4. Materials and Methods

### 4.1. Plant Species and Growth Sites

A controlled-environment experiment was conducted at Oak Ridge National Laboratory (ORNL, Oak Ridge, TN, USA; 35°54′ N, 84°20′ W), using *Liriodendron tulipifera* L. as the material. Two-year-old bare-root saplings were initially established in 6.2 L containers filled with a standard commercial substrate (Sun Gro Horticulture, Agawam, MA, USA) and cultivated outdoors under naturally varying light, temperature, and humidity conditions. Following a ten-month establishment period, saplings exhibiting uniform growth and vigor were selected and repotted into 35-L containers with the identical growth medium. Prior to measurements, all plants underwent a 30-day acclimation period inside a programmable walk-in growth chamber (Model BDW80, Conviron, Winnipeg, MB, Canada). Plants received regular irrigation and were supplemented with a complete 20-10-20 NPK fertilizer (Southern AG, Palmetto, FL, USA) at routine intervals. All experimental measurements were performed in July 2017 across five temperature levels: 25, 30, 35, 40, and 45 °C. The same fully expanded leaves were used for measurements across all five temperature levels. At each target temperature, the chamber was adjusted accordingly, and leaves were allowed a minimum equilibration period of two hours before any recordings were taken. Upon completion of measurements at a particular temperature, the chamber environment was restored to the acclimation baseline. The five temperature treatments were conducted in the order 25, 30, 35, 40, and 45 °C on separate days, allowing plants to recover overnight between successive temperature exposures. Recovery was confirmed by checking that the steady-state photosynthesis returned to its baseline level each morning before the next day’s measurements, ensuring that the photosynthetic apparatus had recovered from the previous day’s heat exposure prior to each new temperature treatment.

### 4.2. Concurrent Measurements of Gas Exchange and Chlorophyll Fluorescence

Gas-exchange and chlorophyll fluorescence measurements were conducted using a LI-6800 portable photosynthesis system (LI-COR Inc., Lincoln, NE, USA) equipped with a multiphase flash fluorometer chamber (6800-01A, LI-COR Inc., Lincoln, NE, USA). Measurements were performed on three individual saplings per treatment, with one fully expanded leaf selected from each sapling, serving as three biological replicates. RH was held constant at 60% with a flow rate of 500 mL min^−1^. Prior to CO_2_-response curve measurements, leaves were light-adapted at the saturating light intensity until steady-state photosynthesis was achieved, a process typically requiring about 20 min. CO_2_-response curves were subsequently measured under light intensity of 1200 μmol m^−2^ s^−1^ across 12 CO_2_ concentrations (400, 300, 200, 150, 100, 50, 500, 650, 800, 1000, 1200, and 1500 μmol mol^−1^). The time interval between measurements at different CO_2_ concentrations was restricted to 120–240 s. Gas exchange parameters (e.g., *A*_n_ and *g*_s_), light-adapted stead-state (*F*_s_) and maximum ($F_{m}^{\prime }$) chlorophyll fluorescence was obtained from CO_2_ response curves for the same leaves. Stomatal conductance to CO_2_ (*g*_s_) was calculated from water vapor conductance (*g*_sw_) using the conversion factor 1.56 (*g*_s_ = *g*_sw_/1.56).

For dark-adapted chlorophyll fluorescence parameters, plants were placed in complete darkness overnight by extinguishing the growth chamber light source prior to measurements. *F*_o_ and *F*_m_ were determined only at 25 °C. *F*_o_ and *F*_m_ at other temperatures (30, 35, 40, and 45 °C) were estimated based on the temperature responses of *F*_o_ and *F*_m_ reported by Pospíšil et al. (1998) [52], which covered all target temperatures (20–45 °C). Note that *F*_o_ and *F*_m_ were measured only at 400 μmol mol^−1^, not across the range of CO_2_ concentrations.

### 4.3. Derivation of Chlorophyll Fluorescence Parameters

The actual PSII photochemical efficiency (*Φ*_PSII_) was determined following the method of Genty et al. (1989) [53]:

(1) $$\Phi_{PSII}=\frac{F_{m}^{\prime }-F_{s}}{F_{m}^{\prime }},$$

$F_{m}^{\prime }$ is Maximum fluorescence in the light-adapted state, *F*_s_ is Steady-state fluorescence in the light-adapted state.

NPQ was determined according to Bilger and Björkman (1990) [54]:

(2) $$NPQ=\frac{F_{m}-F_{m}^{\prime }}{F_{m}^{\prime }},$$

The quantum yields of regulated (*Φ*_NPQ_) and non-regulated (*Φ*_f,D_) energy dissipation in PSII were calculated following Hendrickson et al. (2004) [55]:

(3) $$\Phi_{NPQ}=\frac{F_{s}}{F_{m}^{\prime }}-\frac{F_{s}}{F_{m}},$$

(4) $$\Phi_{f,D}=\frac{F_{s}}{F_{m}},$$

*q*_L_ was calculated according to Kramer et al. (2004) [56]:

(5) $$q_{L}=\frac{F_{m}^{\prime }-F_{s}}{F_{m}^{\prime }-F_{o}^{\prime }}\times \frac{F_{o}^{\prime }}{F_{s}},$$

$F_{o}^{\prime }$ is Minimum fluorescence in the light-adapted state.

*J*_a_ was calculated as:

(6) $$J_{a}=\Phi_{PSII}\times PAR\times \alpha \times \beta ,$$

where PAR denotes the photosynthetically active radiation, *α* is the leaf absorptance coefficient, and *β* is the partition coefficient of absorbed light energy between PSI and PSII. In this study, values of *α* = 0.84 [57,58] and *β* = 0.5 [59] were used consistently for all treatments. We acknowledge that the use of constant *α* and *β* values across the entire temperature range represents a pragmatic simplification. However, given the lack of direct, environment-specific calibrations for each treatment, this approach provides a necessary and widely accepted first-order estimate.

### 4.4. Estimation of Mesophyll Conductance with Variable J Method

*g*_m_ was estimated using the Variable *J* method described by Harley et al. (1992) [60]:

(7) $$g_{m}=\frac{A_{n}}{C_{i}-\frac{\Gamma^{*}(J_{a}+8\left(A_{n}+R_{d}\right))}{J_{a}-4(A_{n}+R_{d})}},$$

where *C*_i_ is the intercellular CO_2_ concentration, *R*_d_ is half of the measured dark respiration rate (*R*_n_, *R*_d_ = *R*_n_/2) [61,62,63]. *R*_n_ was measured after dark adaptation using the LI-6800 system at 25 °C. The temperature dependence of *R*_d_ was corrected according to Bernacchi et al. (2001) [64]:

$$R_{d}=Exp\left(18.72-\frac{46390}{8.314*\left(273.15+T_{leaf}\right)}\right)$$

$\Gamma^{*}$ is the CO_2_ compensation point in the absence of mitochondrial respiration. The temperature dependence of $\Gamma^{*}$ was corrected according to Bernacchi et al. (2002) [18]:

(8) $$\Gamma^{*}=Exp\left(13.49-\frac{24460}{8.314*\left(273.15+T_{leaf}\right)}\right),$$

where *T*_leaf_ is the leaf temperature (°C).

The CO_2_ concentration at the chloroplast carboxylation site (*C*_c_) was calculated as:

(9) $$C_{c}=C_{i}-\frac{A_{n}}{g_{m}},$$

Following the criteria of Harley et al. (1992) [60], we verified that all dC_c_/dA_n_ values remained between 10 and 50, which lends confidence to the validity of our derived g_m_.

### 4.5. Estimation of Mesophyll Conductance and Maximum Carboxylation Rate Using Curve-Fitting Approach

We estimated *g*_m_ and *V*_cmax_ using the curve-fitting method described by Sharkey et al. (2007) [65] and Sharkey (2016) [66], which exploits the curvature of the *A*_n_-*C*_i_ response curve induced by finite *g*_m_. The parameters were obtained by non-linear regression minimizing the sum of squared residuals. To validate the reliability of the estimated *g*_m_ values, we compared the variable *J* method against the *A*_n_/*C*_i_ curve-fitting approach. Both methods were applied to the same dataset, and the results were highly consistent (R^2^ = 0.834, *p* < 0.001; Figure S1). Accordingly, only the *g*_m_ values derived from the variable *J* method are reported herein.

### 4.6. Relative Limitation Analyses

Relative limitation analysis includes *l*_s_, *l*_m_, and *l*_b_. In this study, we followed the approach of Grassi and Magnani (2005) [67] to calculate these components using the following equations:

(10) $$l_{s}=\frac{\frac{g_{tot}}{g_{s}}\times \frac{\partial A_{n}}{\partial C_{c}}}{g_{\mathrm{tot}}+\frac{\partial A_{n}}{\partial C_{c}}}$$

(11) $$l_{m}=\frac{\frac{g_{tot}}{g_{m}}\times \frac{\partial A_{n}}{\partial C_{c}}}{g_{tot}+\frac{\partial A_{n}}{\partial C_{c}}}$$

(12) $$l_{b}=\frac{g_{tot}}{g_{tot}+\frac{\partial A_{n}}{\partial C_{c}}}$$

where *g*_tot_ is the total CO_2_ conductance from ambient air to the chloroplast (1/*g*_tot_ = 1/*g*_s_ + 1/*g*_m_). The slope ∂*A*_n_/∂*C*_c_ was calculated as the first derivative of the *A*_n_/*C*_c_ curve over the *C*_c_ range of 50–100 μmol mol^−1^ for each temperature-CO_2_ combination using linear regression [68].

### 4.7. Data Analyses and Statistics

All statistical analyses and graphics were performed in R version 4.4.2. For graphical presentation, five representative CO_2_ concentrations (150, 300, 500, 650, and 800 μmol mol^−1^) were selected from the full measurement series to clearly illustrate the temperature response trends across sub-ambient to elevated CO_2_ levels. For each treatment, means and standard errors (SE) were calculated from three biological replicates, each from an individual sapling.

Generalized additive mixed models (GAMM) were fitted to the response curves of photosynthetic parameters to temperature or CO_2_ concentration, from which *T*_opt_ for *A*_n_, iWUE, *g*_s_, and *g*_m_ were derived. GAMM were fitted using penalized thin-plate regression splines (bs = “tp”) with *k* = 4. To account for the within-individual correlation arising from repeated measurements on the same leaf across temperatures and CO_2_ treatments, replicate identity (Rep) was included as a random intercept. The models were fitted using restricted maximum likelihood (REML), and gamma = 0.5 was used to increase the penalty on model complexity and reduce the risk of overfitting. Model diagnostics were conducted for each GAMM, including scatterplots of residuals versus fitted values and Q-Q plots to evaluate residual distribution and variance structure. Residual normality was assessed using the Shapiro–Wilk test, and no substantial deviations were found. For each fitted GAMM, we predicted responses over a fine temperature grid and defined *T*_opt_ as the temperature yielding the maximum predicted value. Because *g*_s_ increased again above 40 °C owing to stomatal dysfunction rather than an adaptive response, the *T*_opt_ of *g*_s_ was estimated over the 25–40 °C range; for *A*_n_, iWUE, and *g*_m_, *T*_opt_ was estimated over the full 25–45 °C range. Uncertainty in *T*_opt_ was estimated by parametric bootstrap. For each fitted GAMM, we simulated 1000 sets of response values, refitted the model to each simulated dataset, and re-estimated *T*_opt_ from the fitted curve. The 2.5th and 97.5th percentiles of the resulting *T*_opt_ distribution were taken as the 95% confidence interval.

Subsequent Duncan’s multiple range tests (*p* < 0.05) were performed to identify specific differences among treatments, including (i) comparisons among CO_2_ concentrations within each temperature level, and (ii) comparisons among temperatures within each CO_2_ concentration level. The detailed results, with significance letter assignments for each parameter, are presented in Tables S3 and S4, respectively. To evaluate the effects of temperature, CO_2_ concentration, and their interaction on photosynthetic traits, two-way ANOVA was performed for each parameter, with temperature and CO_2_ concentration as fixed factors.

To further characterize the relationships among physiological traits, correlation analysis was conducted using the measured photosynthetic and chlorophyll fluorescence parameters. Principal component analysis (PCA) was performed to summarize the multivariate variation across temperature and CO_2_ treatments.

## 5. Conclusions and Implications

Our findings reveal that elevated CO_2_ shifts the thermal optima of *g*_m_ and *g*_s_ in opposite directions, decoupling carbon acquisition from water loss. Under elevated CO_2_, plants may close stomata earlier to save water while relying on enhanced mesophyll diffusion to sustain carbon gain below 40 °C, but this strategy fails when *g*_m_ collapses under extreme heat. The shift from stomatal to mesophyll limitation at 45 °C, along with apparent photochemical limitation, implies that current terrestrial biosphere models may overestimate carbon uptake during heatwaves. Many models fail to account for the CO_2_ sensitivity of stomatal conductance parameters, which can lead to significant overestimates of evapotranspiration and carbon uptake under elevated CO_2_ [69]. Moreover, despite being recognized as a key limiting factor in photosynthesis, *g*_m_ is scarcely considered in climate change research [70]. Models should explicitly account for their separate temperature dependencies, including the nonlinear decline of *g*_m_ at high temperatures and temperature-induced declines in electron transport. The distinct responses of *g*_m_ and *g*_s_ also help resolve uncertainties in photosynthetic acclimation, which may involve different mechanisms under ambient versus elevated CO_2_. Finally, the sharp *F*_v_/*F*_m_ decline at 45 °C underscores a fundamental limit to CO_2_ fertilization: once photochemistry is impaired, extra CO_2_ cannot restore carbon gain. Given projections of more frequent heatwaves [33,34], the benefits of elevated CO_2_ may be transient under extreme conditions. Future work should prioritize identifying traits for thermotolerance in photochemistry and mesophyll diffusion and explore acclimation potential in forest trees.

## Figures and Tables

**Table 1:** Optimal temperatures (*T*_opt_) for net photosynthetic rate (*A*_n_), intrinsic water use efficiency (iWUE), stomatal conductance (*g*_s_), and mesophyll conductance (*g*_m_) under different CO_2_ concentrations (*C*_a_) at a photosynthetically Active Radiation (PAR) of 1200 μmol m^−2^ s^−1^. *T*_opt_ for *g*_s_ was estimated over the 25–40 °C range, because *g*_s_ increased again above 40 °C; *T*_opt_ for *A*_n_, iWUE, and *g*_m_ was estimated over the full 25–45 °C range. Values are means with 95% confidence intervals in parentheses. * represents that *T*_opt_ could not be determined within the measured temperature range.

*T*_opt_ (°C)	*C*_a_ (μmol·mol^−1^)				
	150	300	500	650	800
*A*_n_ (μmol·m^−2^·s^−1^)	25.0 (25.0–26.4) *	28.8 (26.7–30.9)	33.4 (31.8–35.1)	34.6 (32.9–36.3)	35.2 (33.2–37.2)
*g*_s_ (mol·m^−2^·s^−1^)	40.0 (38.8–40.0) *	29.9 (28.4–32.3)	30.0 (29.0–31.4)	28.8 (26.2–30.1)	25.0 (25.0–26.5) *
*g*_m_ (mol·m^−2^·s^−1^)	25.0 (25.0–26.4) *	25.0 (25.0–26.4) *	32.7 (31.8–33.7)	34.3 (33.4–35.2)	35.1 (34.2–36.0)
iWUE (μmol CO_2_·mol^−1^ H_2_O)	25.0 (25.0–26.4) *	25.0 (25.0–26.4) *	36.2 (35.2–37.3)	36.7 (35.5–38.0)	36.5 (35.3–37.7)

## Data Availability

The data underlying this article will be shared upon reasonable request to the corresponding author.

## Supplementary Materials

The following supporting information can be downloaded at: https://www.mdpi.com/article/10.3390/plants15182889/s1, Figure S1: Correlation analysis between *g*_m_ Sharkey and *g*_m_ Harley; Table S1: Temperature-dependent changes in vapor pressure deficit (VPD) under the experimental conditions (RH = 60%); Figure S2: Diagnostic plots for the GAMM fitted to the relationship between net photosynthetic rate (*A*_n_) and temperature under 150 μmol mol^−1^ CO_2_; Table S2: Significance analysis of temperature × CO_2_ (*C*_a_) interaction; Table S3: Significance letters for each parameter under different *C*_a_ concentrations at the same temperature, based on Duncan’s multiple range test; Table S4: Significance letters for each parameter under different temperature concentrations at the same *C*_a_, based on Duncan’s multiple range test.

## Abbreviations

The following abbreviations are used in this manuscript:

*A*_n_	Net photosynthetic rate
iWUE	Intrinsic water use efficiency
*g*_s_	Stomatal conductance
*g*_m_	Mesophyll conductance
*T*_opt_	Optimal temperature
*V*_cmax_	Maximum carboxylation rate
*l*_m_	Mesophyll limitation
*l*_s_	Stomatal limitation
*l*_b_	Biochemical limitation
*F*_v_/*F*_m_	Photochemical efficiency
CA	Carbonic anhydrase
VPD	Vapor pressure deficit
*Φ*_PSII_	PSII photochemical efficiency
NPQ	Non-photochemical quenching
*Φ*_NPQ_	Quantum yields of regulated
*Φ*_f,D_	non-regulated energy dissipation in PSII
*q*_L_	Fraction of open reaction centers
*J*_a_	Electron transport rate
*C*_i_	Intercellular CO_2_ concentration
*R*_n_	Net photosynthetic rate
*R*_d_	Day respiration rate
*C*_c_	Chloroplast carboxylation site
*C*_a_	Ambient CO_2_ concentrations
*PAR*	Photosynthetically Active Radiation
*gsc*	Stomatal conductance to CO_2_
*Fo*	Minimum chlorophyll fluorescence
*Fm*	Maximum chlorophyll fluorescence
$F_{m}^{\prime }$	Maximum fluorescence in the light-adapted state
*Fs*	Steady-state fluorescence in the light-adapted state
$F_{o}^{\prime }$	Minimum fluorescence in the light-adapted state
*Tleaf*	Leaf temperature
*Γ**	CO_2_ compensation point in the absence of mitochondrial respiration
*g*_m_/*g*_s_	Ratio of mesophyll to stomatal conductance
*C*_c_/*C*_a_	Ratio of chloroplastic to ambient CO_2_ concentration
*C*_i_/*C*_a_	Ratio of intercellular to ambient CO_2_ concentration
