# Supplementary material for: Elevated CO2 Decouples the Short-Term Thermal Responses of Mesophyll and Stomatal Conductance, Enhancing Carbon Gain and Water Use at Moderate Heat but Failing at Extreme Temperatures
Source: Plants (Basel). 2026 Sep 21;15(18):2889. doi: 10.3390/plants15182889 (PMC13610450; doi:10.3390/plants15182889)
Supplement: Supplementary file 1 [file plants-15-02889-s001.zip › plants-4511855-supplementary.pdf]

Supplementary Material

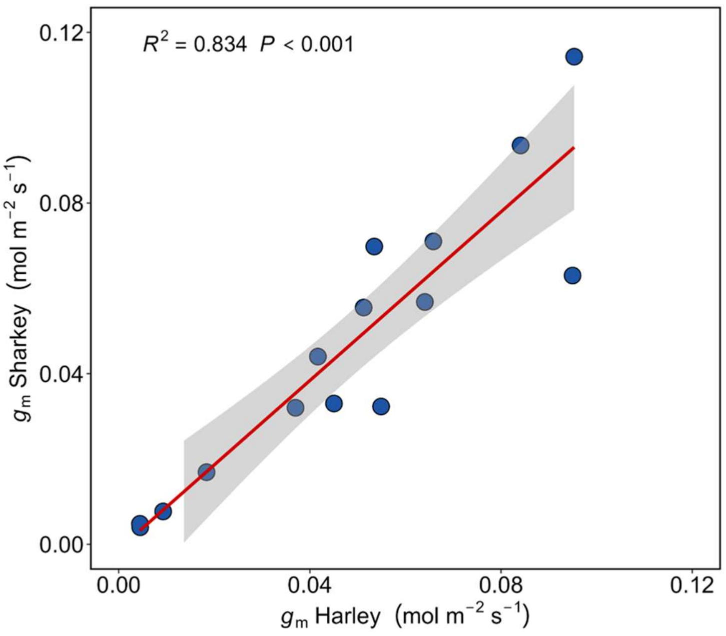

Fig. S1. Correlation analysis between g_m Sharkey and g_m Harley. g_m Sharkey was derived from fitting the A_n–C_c curve according to Sharkey's method, while g_m Harley was calculated using Harley's variable J method.

Table S1. Temperature-dependent changes in vapor pressure deficit (VPD) under the experimental conditions (RH = 60%).

Temperature (°C)	VPD (kPa)
25	1.37
30	1.74
35	2.27
40	2.84
45	3.32

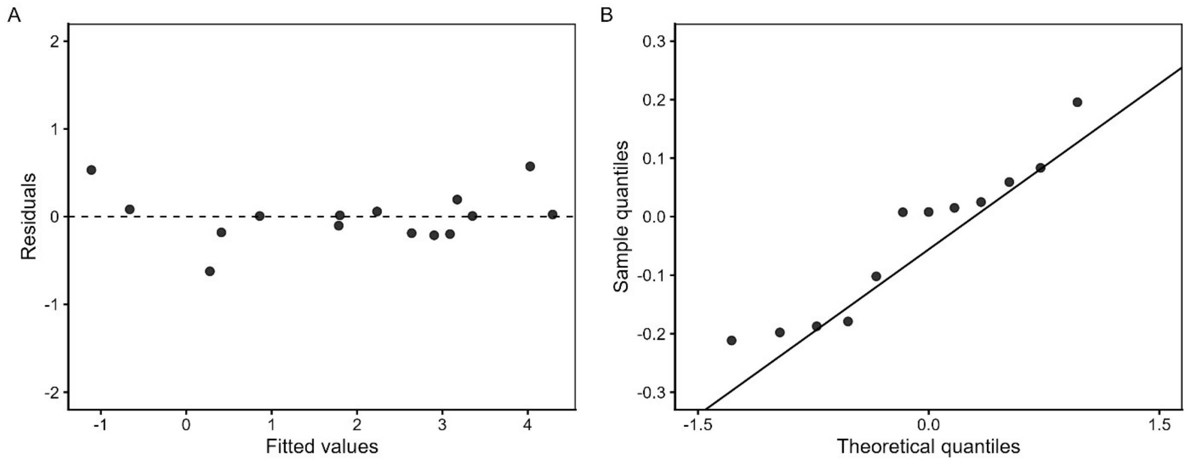

Figure S2. Diagnostic plots for the GAMM fitted to the relationship between net photosynthetic rate (A_n) and temperature under 150 μmol mol^{-1} CO_2. (A) Residuals versus fitted values; (B) Normal Q–Q plot.

Table S2. Significance analysis of temperature × CO_2 (C_a) interaction.

Parameter	*F*	*P*
A_n (μmol·m^{-2}·s^{-1})	11.79	<0.05
g_s (mol·m^{-2}·s^{-1})	4.51	0.051
g_m (mol·m^{-2}·s^{-1})	10.47	<0.05
iWUE (μmol CO_2·mol^{-1} H_2O)	5.17	<0.05
g_m/g_s	8.18	<0.05
C_c/C_a	106.07	<0.05
C_i/C_a	9.51	<0.05
J_a (μmol·m^{-2}·s^{-1})	10.92	<0.05
NPQ	6.47	<0.05
q_L	14.10	<0.05
l_s	7.25	<0.05
l_m	10.37	<0.05
l_b	21.00	<0.05

F: test statistic for between-group differences; *P*: probability of observing the current results under the null hypothesis.

Table S3 Significance letters for each parameter under different C_a concentrations at the same temperature, based on Duncan's multiple range test.

Tempeature (°C)	C_a (μmol mol^{-1})	A_n (μmol·m^{-2}·s^{-1})	g_s (mol·m^{-2}·s^{-1})	g_m (mol·m^{-2}·s^{-1})	iWUE (μmol CO_2·mol^{-1} H_2O)	g_m/g_s	C_c/C_a	C_i/C_a	J_a (μmol·m^{-2}·s^{-1})	NPQ	q_L	l_s	l_m	l_b
25	150	d	b	a	d	a	a	c	a	a	b	a	c	b
25	300	c	b	a	c	a	ab	d	a	a	a	a	d	ab
25	500	b	a	b	bc	b	ab	b	a	a	a	b	b	a
25	650	ab	a	b	ab	b	b	ab	a	a	a	bc	a	a
25	800	a	a	b	a	b	b	a	a	a	a	c	a	a
30	150	d	b	ab	c	b	a	a	b	a	b	a	c	d
30	300	c	b	a	b	a	b	b	ab	a	a	a	d	b
30	500	b	a	bc	b	c	b	a	a	a	a	b	b	a
30	650	ab	a	c	ab	c	b	a	a	a	a	b	ab	ab
30	800	a	b	c	a	c	b	a	a	a	a	b	a	c
35	150	d	ab	ab	d	a	a	a	c	a	c	a	a	b
35	300	c	bc	a	cd	a	b	b	b	a	b	a	a	ab
35	500	b	a	ab	bc	a	c	b	a	b	a	a	a	a
35	650	ab	abc	ab	ab	a	cd	b	a	b	a	a	a	ab
35	800	a	c	b	a	a	d	b	a	b	a	a	a	b
40	150	c	a	b	d	a	a	a	d	a	d	a	a	c
40	300	c	b	a	cd	a	b	b	cd	a	c	a	b	b
40	500	b	a	a	bc	a	c	b	bc	ab	b	a	b	a
40	650	ab	ab	a	ab	a	d	b	ab	b	a	a	b	ab
40	800	a	b	ab	a	a	d	b	a	b	a	a	b	b
45	150	d	d	a	b	a	a	a	c	d	b	a	c	a
45	300	c	e	d	ab	b	b	b	b	e	a	b	a	c
45	500	b	a	bc	ab	b	c	b	ab	c	a	b	b	b
45	650	ab	b	b	a	b	c	b	ab	b	a	b	b	b
45	800	a	c	cd	a	b	d	b	a	a	a	b	ab	bc

Table S4 Significance letters for each parameter under different temperature concentrations at the same C_a, based on Duncan's multiple range test.

C_a (μmol mol^{-1})	Temperature (°C)	A_n (μmol·m^{-2}·s^{-1})	g_s (mol·m^{-2}·s^{-1})	g_m (mol·m^{-2}·s^{-1})	iWUE (μmol CO_2·mol^{-1} H_2O)	g_m/g_s	C_c/C_a	C_i/C_a	J_a (μmol·m^{-2}·s^{-1})	F_v/F_m	NPQ	q_L	l_s	l_m	l_b
150	25	a	a	a	a	a	d	e	c	a	a	c	a	bc	b
150	30	a	a	a	b	ab	cd	d	ab	a	b	b	ab	bc	b
150	35	b	a	a	c	b	bc	c	a	a	bc	a	b	b	b
150	40	c	a	b	d	c	b	b	bc	b	bc	a	c	a	b
150	45	d	a	b	e	c	a	a	d	c	c	c	d	c	a
300	25	a	a	a	a	a	b	c	c	a	a	bc	a	c	a
300	30	a	a	a	a	ab	b	c	ab	a	b	b	a	c	a
300	35	a	a	ab	a	ab	b	c	a	a	b	a	a	c	a
300	40	b	a	b	b	b	b	b	bc	b	b	a	a	b	a
300	45	c	a	c	c	c	a	a	d	c	b	c	b	a	a
500	25	ab	a	ab	a	a	b	b	c	a	a	b	a	c	ab
500	30	a	a	ab	a	a	b	b	b	a	b	b	a	bc	ab
500	35	a	a	a	a	a	b	b	a	a	c	a	a	bc	bc
500	40	b	a	b	a	a	b	b	ab	b	bc	a	a	ab	c
500	45	c	a	c	b	b	a	a	d	c	bc	b	b	a	a
650	25	a	a	b	a	ab	b	b	c	a	a	b	a	a	b
650	30	a	a	ab	a	ab	b	bc	bc	a	b	b	a	a	b
650	35	a	a	a	a	a	b	c	a	a	c	a	a	a	bc
650	40	a	a	b	a	a	b	bc	ab	b	c	a	a	a	c
650	45	b	a	c	b	b	a	a	d	c	b	b	b	a	a
800	25	a	a	b	ab	ab	ab	ab	b	a	a	c	bc	ab	a
800	30	a	a	ab	a	ab	bc	bc	b	a	a	c	ab	ab	b
800	35	a	a	a	a	a	c	c	a	a	b	b	a	b	bc
800	40	a	a	ab	a	a	c	c	a	b	b	a	ab	ab	c
800	45	b	a	c	b	b	a	a	c	c	a	c	c	a	a
